# Prevention of deep infection in joint replacement surgery: A review

**DOI:** 10.3109/17453674.2010.537805

**Published:** 2010-11-26

**Authors:** Esa Jämsen, Ove Furnes, Lars B Engesæter, Yrjö T Konttinen, Anders Odgaard, Anna Stefánsdóttir, Lars Lidgren

**Affiliations:** ^1^Coxa, Hospital for Joint Replacement, Tampere; ^2^Geriatric Unit, Seinäjoki Central Hospital, Seinäjoki, Finland; ^3^Department of Orthopedic Surgery, Haukeland University Hospital, Bergen; ^4^Department of Surgical Sciences, University of Bergen, Norway; ^5^Department of Medicine, Helsinki University Central Hospital, Helsinki, Finland; ^6^Department of Orthopedics, Aarhus University Hospital, Aarhus, Denmark; ^7^Department of Orthopedics, Clinical Sciences, Lund University, Lund, Sweden

The incidence of deep infection has declined since the early years of joint replacement surgery (Figure 1). Currently, the infection rates are low: around 1% in primary knee replacements and 0.3–0.6% in hip replacements (Phillips et al. 2006, Pulido et al. 2008, Jämsen et al. 2010). However, even prospective surveillance programs may underestimate the infection rates; thus, the true incidence is probably higher (Huotari et al. 2010).

**Figure 1.**
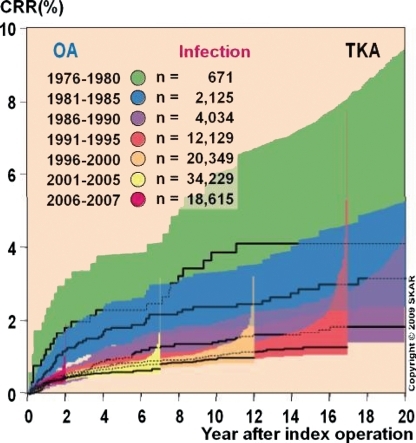
The cumulative revision rates (CRRs; the proportion of operated patients who underwent revision with time) with revision for infection as endpoint in consecutive cohorts of primary total knee arthroplasties (TKAs) performed in patients with osteoarthritis (OA) in 1976–2007 in Sweden. The colored areas represent the 95% confidence intervals for the cumulative revision rates for different time periods. Source: the Swedish Knee Arthroplasty Register, 2009.

Deep infection accounts for up to one quarter of early revisions (Dobzyniak et al. 2006, Mulhall et al. 2006). Recent data from the Scandinavian arthroplasty registries show that the proportion of revision operations that are due to infection is increasing (Figure 2). Operating patients with a higher inherent infection risk, such as obese patients and those with diabetes, and emergence of resistant bacterial strains represent additional challenges, and give reason for continuous dedication to prevent deep infection.

**Figure 2.**
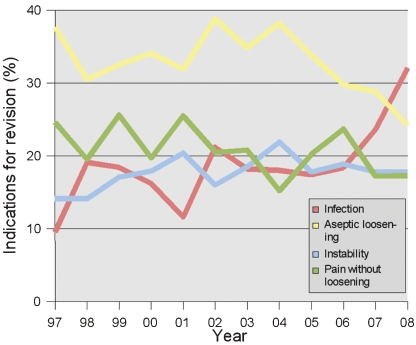
The proportions of infections, aseptic loosening, instability, and pain for all reasons for revision knee replacements in 1997–2008 in Denmark. Source: the Danish Knee Arthroplasty Register, Annual Report 2009 (available online at www.dkar.dk).

Patient-related infection risk can be reduced e.g. by managing preoperative anemia, glucose control, and elimination of harmful lifestyle factors such as smoking. Morbidly obese patients represent a special risk group. The principles of infection prevention in perioperative management are well-documented, but adherence to the protocols should be improved. Care should be taken regarding timely and appropriate administration of antibiotic prophylaxis. Combining intravenous antibiotics and antibiotic-impregnated cement further reduces deep infection rates. Finally, monitoring of infection rates on a local, national, and even an international scale is an essential part of quality control and is necessary in order to be able to identify weaknesses in current infection prevention practices.

## Pathogenesis

Deep postoperative infection is traditionally classified into early infection (< 3 months postoperatively), delayed infection (3–24 months), and late infection (> 2 years postoperatively) (Zimmerli et al. 2004). Approximately one third of deep infections occur within 3 months and two-thirds within 2 years after the index operation (Phillips et al. 2006, Jämsen et al. 2009b, Stefánsdóttir et al. 2009a). Hematogenous infection may occur at any time after the operation, but its proportion increases with time after surgery. Overall, hematogenous infections account for up to almost one third of infected joint replacements (Pulido et al. 2008, Stefánsdóttir et al. 2009a).

Most of the relevant literature deals with early and delayed postoperative infections where the infecting pathogen is thought to contaminate the joint during surgery (Zimmerli et al. 2004). These infections are potentially preventable by minimizing the possibility of perioperative and early postoperative contamination of the prosthesis.

## Patient-related risk of deep infection

In general, any co-morbid condition that impairs host defense mechanisms, prolongs wound healing, or predisposes to wound-related complications should be considered a potential risk factor for deep infection. In knee replacements, there is good evidence of higher risk of deep infection in patients with rheumatoid arthritis (RA), American Society of Anesthesiologists (ASA) risk score > 2, diabetes, or morbid obesity (Jämsen 2009). Most risk factors are shared for knee and hip replacements (Pulido et al. 2008).

Patients with RA have a higher risk of deep infection (Schrama et al. 2010). Biological anti-rheumatic drugs in particular may predispose to wound infections (Konttinen et al. 2005). Other inflammatory arthritides do not appear to be associated with increased infection risk after primary knee replacement (Jämsen et al. 2009a). Secondary knee osteoarthritis, earlier fractures, and acute hip fractures especially are also associated with increased risk of deep infection after joint replacement (Ridgeway et al. 2005, Jämsen 2009). In knee replacements, poorer preoperative Knee 55 Society Scores asssociate with higher risk of deep infection (Jämsen et al. 2010). The infection rates are higher in revisions than in primary hip and knee replacements.

ASA risk index is used as a proxy for co-morbidity. The risk of deep infection and other postoperative complications increases almost exponentially with ASA score. However, cardiovascular, respiratory, and neurological disorders do not appear to result in higher infection rate, even though they increase the ASA risk score (Peersman et al. 2001). Accordingly, in the study by Lai et al. (2007), only diabetes and urogenital disorders were associated with infected joint replacements. Preoperative anemia is predictive of a need for allogenic blood transfusions, which in turn have been associated with higher risk of postoperative infections (Pulido et al. 2008).

The role of obesity and diabetes should be acknowledged when preventing deep infections. Obesity is more common in knee replacement recipients than in hip replacement recipients (Fehring et al. 2007). The highest infection rates have been observed in morbidly obese patients (body mass index ≥ 40 kg/m^2^) (Pulido et al. 2008, Dowsey and Choong 2009, Jämsen 2009). Wound healing problems, including wound infection, are common in this patient group but these patients also often have underlying conditions such as diabetes or peripheral vascular disease that may give few symptoms but nevertheless increase the risk of infection.

Meding et al. (2007) identified diabetes preoperatively in 15% of joint replacement recipients, only 58% of whom were previously diagnosed. Since diabetes increases the risk of surgical site infections (Mangram et al. 1999), screening for diabetes might be a reasonable approach (Jämsen 2009). There is preliminary evidence to suggest that patients with poorly controlled diabetes have a higher risk of postoperative complications (Marchant et al. 2009), including infection (Jämsen 2009).

Malnutrition and smoking delay wound healing and increase the risk of infection (Mangram et al. 1999), as does alcohol abuse (Peersman et al. 2001). In contrast to RA, for example, these lifestyle-related risk factors, together with obesity and glycemic control, are potentially modifiable risk factors.

Smoking should be discontinued 6–8 weeks before surgery. In a randomized study, participation in a preoperative smoking cessation program was found to reduce postoperative complication rates, and no wound-related complications occurred in the patients who stopped smoking before surgery (Møller et al. 2002). In an experimental study, use of transdermal nicotine patches did not impair wound healing (Sørensen et al. 2003).

## Optimizing the patient's condition preoperatively

All current infections should be managed before the operation. The most common sources of hematogenous infection are the skin, and the urinary and respiratory tracts. With hip or knee replacement, the skin of the lower extremities should be intact. Treatment of asymptomatic bacteriuria, which is common in elderly patients, preoperatively is not necessary, whereas symptomatic urinary tract infection should probably be treated (David and Vrahas 2000). Major dental procedures are a potential source of hematogenous infection—although the underlying evidence is weak (Uçkay et al. 2008)—and it is advisable to perform such operations before joint replacement when possible.

Nasal carriage of *Staphylococcus aureus*, including methicillin-resistant strains, increases the risk of surgical site infections (Mangram et al. 1999, Yano et al. 2009). Although preoperative treatment using nasal mupirocin ointment reduces the risk of nosocomial *S. aureus* infections, it has not been shown to reduce the risk of surgical site infections in patients with nasal *S. aureus* carriage (van Rijen et al. 2008).

## Perioperative management

The sterile techniques and measures thoroughly described by the Centers for Disease Control and Prevention (Mangram et al. 1999) should be also used as a routine in joint replacement surgery, but the effectiveness of all of these techniques has not been evaluated in this field of surgery.

The duration of preoperative hospitalization should be minimized to reduce the risk of colonization of the patient's skin with possibly resistant hospital-acquired bacterial strains. Arrival on the day of operation is becoming a routine.

Antiseptic agents do not appear to differ much regarding postoperative infection rates (Edwards et al. 2009), but the long-acting chlorhexidine is favored (Fletcher et al. 2007). Preoperative bathing, showering using antiseptic agents, or use of plastic adhesive drapes has not been found to reduce the risk of postoperative infections (Webster and Osborne 2007, Webster and Alghamdi 2009). Hair removal, if done at all, should be done immediately preoperatively and using clippers or depilatory agents (Mangram et al. 1999, Fletcher et al. 2007).

The effectiveness of different solutions for hand washing probably depends not only on their antiseptic activity but also on the surgeons' compliance, scrubbing technique, and duration of the scrub (Mangram et al. 1999). Surgical gowns, masks and caps, and personal exhaust systems reduce bacterial counts in the air of the operating room but it is unclear whether different kinds of surgical attire can affect postoperative infection rates (Mangram et al. 1999, Fletcher et al. 2007). Double-gloving and use of indicator gloves probably reduce the risk of hand contact between the surgeon and the patient, and can be recommended.

Hypothermia and dehydration may impair microcirculation in the operating field and thereby weaken host defense mechanisms (Mangram et al. 1999). Maintenance of normothermia during the operation has resulted in lower rates of postoperative infection in general and abdominal surgery (Forbes et al. 2009), but there has been a lack of such studies in joint replacement surgery. Sufficient oxygenation may also be of importance (Mangram et al. 1999).

Surgical stress induces insulin resistance; this leads to a catabolic state and hyperglycemia, which may persist for weeks postoperatively and predispose the patient to wound-related complications (Ljungqvist et al. 2007). Importantly, this may also occur in non-diabetic patients. In thoracic surgery, postoperative hyperglycemia increases the risk of mediastinitis, and conversely, strict glycemic control postoperatively has resulted in lower infection rates (Furnary and Wu 2006). Although perioperative hyperglycemia occurs in up to three quarters of non-diabetic patients undergoing knee or hip replacement (Pili-Floury et al. 2009), there have been no studies evaluating the association between perioperative hyperglycemia and postoperative infection rate in the field of joint replacement surgery.

Larger constrained and especially hinged prostheses used in the management of severe knee deformities carry a higher risk of infections than non-constrained TKR (Jämsen et al. 2009a). Unicompartmental knees (UKR) have lower infection rates (approximately one third of that following total knee replacement) (Furnes et al. 2007, Swedish Knee Arthroplasty Register 2009). Prolonged operating time (> 2.5 h)—reflecting the complexity of surgery or the inexperience of the surgeon—has been associated with increased infection rate in several studies (Peersman et al. 2001, Gastmeier et al. 2005, Pulido et al. 2008, Dale et al. 2009, Jämsen et al. 2010).

Prompt surgical management of wound-related problems is advocated by some authors, but there have been no studies comparing early aggressive management vs. nonoperative management in clinical practice. In a large retrospective review, revision and deep infection rates were found to be high also after early surgical management of wound-healing problems, but the study lacked a comparison group with nonoperatively treated patients (Galat et al. 2009).

Thromboprophylaxis increases the risk of hematoma and consequent wound-related problems. Preoperative administration of low-molecular-weight heparin was found to be associated with more infected knee replacements in a case-control study (Asensio et al. 2005). Data from prospective trials is lacking, since surgical site infections have not been analyzed as outcome in the existing randomized trials.

Closed suction drains are a potential entry point of infection, but do not seem to affect the wound infection rate (Parker et al. 2007). Preliminary results suggest that intra-articular catheters used for administering analgesics postoperatively may also predispose to wound contamination and infection (Reeves and Skinner 2009).

## Antibiotic prophylaxis

Systemic intravenous antibiotic prophylaxis reduces the risk of postoperative infections (AlBuhairan et al. 2008). Cephalosporins are widely used, based on their good efficacy against staphylococcal species and uropathogens (Mangram et al. 1999). Vancomycin is indicated in high-risk patients carrying methicillin-resistant *Staphylococcus aureus* (Mangram et al. 1999, Fletcher et al. 2007, Scottish Intercollegiate Guidelines Network 2008). If the patient has allergy to beta-lactam antibiotics, clindamycin or vancomycin can be used.

The association between time of administration of the antibiotic and surgical site infection rate can be presented as a U-shaped curve with higher risk of infection both before and after the optimal time frame of administration. In a recent large study, the lowest infection rates were seen when administration occurred between 30–60 min before incision (Weber et al. 2008) whereas in another study focusing on total hip replacements, administration within 30 min before incision resulted in the lowest infection rate (van Kasteren et al. 2007). The risk of infection is particularly high if administration occurs after the incision. In both of these studies, cefuroxime or cefazolin was used most frequently. With other antibiotics, the optimal time for administration may differ, depending on the pharmacokinetics. Nevertheless, in knee replacements the antibiotic infusion should be finished at least 10 min before application of a tourniquet (Tomita and Motokawa 2007).

The studies and guidelines concerning duration of routine antibiotic prophylaxis have come to varying conclusions (Engesæter et al. 2003, van Kasteren et al. 2007, Scottish Intercollegiate Guidelines Network 2008). In a large Norwegian register study, 4 doses of intravenous antibiotics on the day of operation were more effective than fewer doses in primary hip replacements (Engesæter et al. 2003). Others have reported no difference (van Kasteren et al. 2007). In prolonged surgeries—including bilateral operations—additional doses may be required, especially when an antibiotic with a short half-life is used (Stefánsdóttir et al. 2009b).

**Figure 3.**
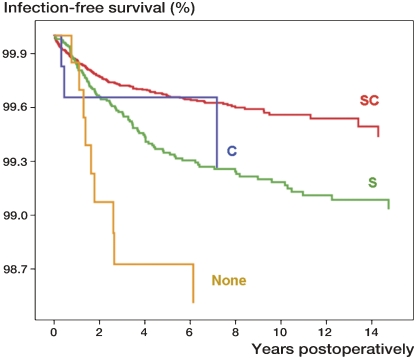
Prosthesis survival with revision due to infection as endpoint following 45,250 primary total hip replacements, performed in Norway in 1987–2007, where no antibiotic prophylaxis (None), intravenous antibiotic prophylaxis (S), antibiotic-impregnated cement (C), or both intravenous antibiotic prophylaxis and antibiotic-impregnated cement (SC) was used. 2,137 operations were performed in a clean-air enclosure, 21,627 in operating theaters with laminar flow, and the remaining operations in operating theaters with standard air ventilation.

In Scandinavia, antibiotic-impregnated cement is also used routinely in primary joint replacements (Engesæter et al. 2003, Jämsen et al. 2009a), although this practice has been questioned and the scientific data from randomized studies are controversial (van de Belt et al. 2001). The basis for this practice comes largely from registry-based studies on hip replacements. Initially, a Norwegian Arthroplasty Register study showed that combining antibiotics intravenously and in cement (combined antibiotic prophylaxis) was more effective that either technique alone in lowering deep infection rates (Espehaug et al. 1997). The result was repeated in a larger series followed for up to 16 years (Engesæter et al. 2003) (Figure 3). In a recent large Norwegian register study, uncemented hip replacements had an overall 1.4-fold risk of revision for infection, compared to hip prostheses fixed with antibiotic-impregnated cement (Dale et al. 2009). Similar results concerning the effect of antibiotic-impregnated cement in primary and revision knee replacements were reported in a Finnish study (Jämsen et al. 2009a) and by the Australian National Joint Registry (Australian Orthopaedic Association 2009).

Emphasizing the value of combined antibiotic prophylaxis in high-risk operations, Chiu et al. have shown in a series of randomized studies that combining antibiotic-impregnated cement and systemic antibiotic prophylaxis prevents deep infection in knee replacements performed under suboptimal operative conditions (Chiu et al. 2002) and with a high baseline risk of infection (patients with diabetes, revision knee replacements) (Chiu et al. 2001, Chiu and Lin 2009).

Antibiotic-impregnated cement has been thought to increase the risk of allergic reactions, emergence of resistant bacterial strains, and aseptic loosening of the prosthesis, but to date there is little evidence to support these concerns. Registry-based studies can be criticized, as provider- and patient-related factors not recorded in the arthroplasty registers may affect the results. However, randomized studies on the effectiveness of antibiotic-impregnated cement are not feasible for ethical, practical, and statistical reasons.

## Provider-related issues

The lowest infection rates have been reported from specialized orthopedic units with high annual operation volumes (Peersman et al. 2001, Phillips et al. 2006, Pulido et al. 2008, Jämsen et al. 2010). In joint replacement surgery, the associations between operation volume and short-term outcomes have been based mostly on administrative health register data, such as Medicare databases (Shervin et al. 2007).

Most studies concerning the effect of hospital volume have found no association between volume and infection rate (Shervin et al. 2007). In a recent study using arthroplasty register data, the type of operating hospital (university, central, district, or other) and hospital annual operation volume was not associated with the rate of infection at medium-term follow-up (Jämsen 2009).

At the surgeon level, a positive association between operation volume and infection rates seems clearer. The published studies, including one using prospectively collected infection surveillance data, suggest that surgeons should perform at least 20–50 joint replacement operations per year (Muilwijk et al. 2007, Shervin et al. 2007). In larger units, it is advisable that joint replacement surgery should be centralized to a smaller group of surgeons.

Clean-air measures and vertical laminar flow have been thought to lead to lower infection rates (Lidwell et al. 1987, Mangram et al. 1999). These early results have been questioned in more recent series where intravenous antibiotic prophylaxis has been used routinely (Brandt et al. 2009). However, in a post hoc analysis Lidwell et al. (1987) showed that combining all available techniques (laminar air flow, body exhaust suits or surgical enclosures, and antibiotic prophylaxis) resulted in the lowest infection rate—of only 0.1%. Antibiotics appear to be most cost-effective, and supplementary use of other prophylactic techniques (antibiotic-impregnated cement, body exhaust suits, and sterile surgical enclosures) increases the cost of prophylaxis—but on the other hand reduces the infection rate (Persson et al. 1999).

The degree of adherence to infection control guidelines is often low in clinical practice. Timely inappropriate administration of antibiotic prophylaxis occurs in 13–50% of cases (Bedouch et al. 2004, Stefánsdóttir et al. 2009b). Selection, dosing, and duration of antibiotic prophylaxis has also been reported to vary (Bedouch et al. 2004, Stefánsdóttir et al. 2009b).

Monitoring of infection rates is important for identification of inappropriate practices, and systematic prospective surveillance has been shown to reduce infection rates (Mangram et al. 1999, Gastmeier et al. 2005). Personnel-related factors (which partly explain the deficiencies) can be minimized using checklists and reminders and decision support, which have both been shown to result in lower rates of surgical site infection (Webb et al. 2006, Haynes et al. 2009). Evidence showing that strict adherence results in fewer infections is lacking. Nevertheless, it has been estimated that 20% of hospital-acquired infections can be prevented by improving adherence to infection control measures (Harbarth et al. 2003).

## Arthroplasty registers in the study of infected joint replacement

Large series of patients are required to study the factors associated with infected joint replacement, because the infection rates are low. Such materials cannot be collected easily in individual hospitals. Thus, arthroplasty registries are necessary in this field of research. However, these registries underestimate the true incidence of deep infections, as they record reliably only revision joint replacements (Jämsen et al. 2009b). As early surgery with prosthesis retention is becoming more popular for acute infections, incomplete registration is of increasing relevance.

In addition, the registries do not have a uniform definition of infection and the validity of diagnosis of infections in arthroplasty registry data relies on the reports made by operating surgeons and reporting activity. Without microbiological confirmation, the diagnosis remains uncertain. Finally, the data concerning patient-related risk factors is in most cases insufficient (i.e. data on co-morbid diseases). Although more patient-related data could be recorded by registries, this might make registration and reporting of data more difficult and have a negative effect on the quality and coverage of data.

Using data from other electronic sources is one way to improve validity. Reoperations other than revision joint replacements (e.g. debridements and partial revisions) can be detected using hospital discharge data from patient registries. Patient registry data can also be used to analyze the effects of co-morbid diseases in more detail. Regional and national infection surveillance programs have been established in some countries, and act as a potential source of microbiological data—although their follow-up period is usually restricted to 1 year (Mangram et al. 1999, Huotari et al. 2010).

Thus, registry-based analyses cannot fully replace clinical studies, but they are still an invaluable source for hypotheses generation, for identification of case patients to be evaluated in more detail (see e.g. Stefánsdóttir et al. 2009a, b), and for quality control.
